# Supplementary material for: The amount of membrane cholesterol required for robust cell adhesion and proliferation in serum-free condition
Source: PLoS One. 2022 Jul 20;17(7):e0259482. doi: 10.1371/journal.pone.0259482 (PMC9299302; doi:10.1371/journal.pone.0259482)
Supplement: S1 Table — Cq value for Fig 3B (upper) and Fig 4B (bottom). The expression of cholesterol biosynthesis-related genes as genes of interest is measured between cultured cells in each condition as biological groups. Each biological group contains three biological replicates. b-ACTIN is used as a reference gene. (PDF) [file pone.0259482.s001.pdf]

Supporting Information

		DF10			RPMI-G			DA-X		
		Sample #1	#2	#3	Sample #1	#2	#3	Sample #1	#2	#3
Target Genes	*SREBF1*	27.92	28.06	28.11	25.85	25.75	25.68	27.86	27.90	27.93
	β-ACTIN	17.42	17.19	18.03	16.40	16.24	16.23	18.07	18.07	18.06
	SREBF2	23.66	23.59	23.53	22.05	22.09	21.87	25.07	25.07	25.17
	β-ACTIN	17.64	17.22	17.24	16.40	16.34	16.13	18.07	18.08	18.06
	ACSS2	26.28	28.07	27.67	26.36	26.28	24.02	27.88	27.73	27.82
	β-ACTIN	16.26	17.22	17.24	19.30	19.35	17.77	17.69	17.34	17.31
	HMGCR	24.60	24.59	24.83	21.82	21.65	21.74	24.76	24.85	24.93
	β-ACTIN	17.64	18.01	17.22	16.05	16.13	16.09	18.04	18.08	18.12
	HMGCS1	22.67	22.78	22.77	19.52	19.78	19.63	24.07	24.06	23.29
	β-ACTIN	17.64	18.01	18.03	16.13	16.24	16.23	18.07	18.08	18.06
	LDLR	23.61	24.47	23.47	22.39	21.79	22.24	25.21	24.59	25.22
	β-ACTIN	17.24	18.03	17.19	16.40	16.09	16.34	18.07	18.08	18.06
	ACLY	23.05	23.81	22.91	22.05	21.67	22.08	25.12	24.80	25.09
	β-ACTIN	17.24	18.03	17.19	16.40	16.09	16.34	18.07	18.08	18.06

		Control			RGDS peptide		
		Sample #1	#2	#3	#1	#2	#3
Target Genes	*SREBF1*	24.26	24.87	24.47	24.63	24.80	24.60
	β-ACTIN	14.17	14.56	14.36	14.77	15.14	14.56
	SREBF2	21.99	22.53	22.48	22.56	22.39	22.46
	β-ACTIN	14.17	14.78	14.55	15.12	15.20	15.17
	ACSS2	21.32	21.37	21.35	22.45	22.40	22.47
	β-ACTIN	14.17	14.12	14.15	15.17	15.12	15.00
	HMGCR	22.02	22.05	22.07	22.21	22.19	22.23
	β-ACTIN	14.56	14.54	14.73	15.17	15.12	15.21
	HMGCS1	20.46	20.40	20.42	20.61	20.43	20.44
	β-ACTIN	14.56	14.54	14.55	15.17	14.77	14.76
	LDLR	21.55	21.64	21.81	22.02	22.02	22.02
	β-ACTIN	14.38	14.56	14.66	15.20	15.19	15.21
	ACLY	23.54	20.55	23.60	23.99	24.01	23.99
	β-ACTIN	16.98	14.22	17.01	17.79	17.79	17.79
