# Supplementary material for: The amount of membrane cholesterol required for robust cell adhesion and proliferation in serum-free condition
Source: PLoS One. 2022 Jul 20;17(7):e0259482. doi: 10.1371/journal.pone.0259482 (PMC9299302; doi:10.1371/journal.pone.0259482)
Supplement: Attachment — Submitted filename: Response to Reviewers.doc [file pone.0259482.s002.doc]

I have separated my responses to the reviewers’ comments according to each reviewer.

---------------------------------------------------------------------------------------------------

*Reviewer #1:*

*While this is an interesting finding, there are some points that should be addressed before this research article is consider for publication.*

*Major points:*

1- *In line 169 it is stated that almost all cells detached and underwent apoptosis within 48 hours (Fig. 2a, two images at bottom) after the use of the selective inhibitor of Oxidosqualene cyclase, although there are no assessments of apoptosis.*
2- *Since the authors did not conduct a concentrations dependent experiment on Ro48-8071, toxicity of the compound cannot be excluded. It has been previously shown the effect of Ro48-8071 in (nM), therefore, I will recommend to perform a concentrations dependent experiment on Ro48-8071 with maximum concentration of 1 μM on cells cultured in DA-X condition. Authors may include the results in a supplementary document and revise the main results accordingly, if needed.*

Our responses to Major points:

1- We fully agree with you and have incorporated this suggestion. In our revised manuscript, we described it as “cell death”, not “apoptosis” in line 171 of “Manuscript” file. Thank you so much for your suggestion.
2- You have raised an important question regarding “toxicity of the compound”; since we’re sorry that this part was not clear in the original manuscript. However, we believe that it can be excluded from the conclusion since the harmful effects of Ro48-8071 even at 1µM on HeLa cells cultured in 10%FBS/DMEM were not shown at all in figure 2a (two images at the top). Therefore, we should have explained that “Ro48-8071 known as a cholesterol biosynthesis inhibition by selective inhibition of Oxidosqualene cyclase had no harmful effects at all on adherent HeLa cells cultured in 10% FBS/ DMEM medium.” in line 167-169 of “Manuscript” file, as we have revised the contents of this part. We would like to express our thanks to give us an opportunity to reconsider the possibility of the compound toxicity.

*Minor points:*

*1- In line 106, reference is missing.*

*2- Correct the spelling of “Oxidosqualen cyclase” to Oxidosqualene cyclase.*

3- *Enhance the resolution of the histograms in figure 3b and 4b, so the entire information can be easily seen.*

Our responses to Minor points:

1- We have incorporated your comments by the addition of an appropriate reference in line 106. In addition, we realized with your comment to need an appropriate reference regarding the function of Methyl-β-cyclodextrin and so made it in line 166 of “Manuscript” file. Thank you very much for your suggestion.
2- We have corrected spelling of this throughout our revised paper (in line 106 and 168 of “Manuscript” file). Thank you very much for pointing out our mistakes.
3- By checking the files of figure at submission, the resolution of the histograms in figure 3b and 4b was high enough to clearly see the information. This is probably due to the conversion of the image file format when the manuscript was compiled for sending to reviewers. Therefore, we believe that sufficient resolution of the histograms can be ensured for publication.

---------------------------------------------------------------------------------------------------

*Reviewer #2: The manuscript "The amount range of membrane cholesterol required for robust cell adhesion and proliferation in serum-free condition by Takii et al. is well-studied and well-written. I recommend for publication.*

*Minor comments*

*1. The title is not conclusive and if "range" is removed, the title would be better received. or The title can be modified for better and as conclusive.*

Our responses to Minor points:

1- We fully agree with you and have incorporated this suggestion by removing the word “range” from the title (in line 2 of “Manuscript” file). I would like to express our thanks for allowing us an opportunity to reconsider the title for more conclusive.

---------------------------------------------------------------------------------------------------
